# Century‐long stomatal density record of the nitrophyte, *Rubus spectabilis* L., from the Pacific Northwest indicates no effect of changing atmospheric carbon dioxide but a strong response to nutrient subsidy

**DOI:** 10.1002/ece3.8405

**Published:** 2021-12-01

**Authors:** Ron Ydenberg, Ben Leyland, Mark Hipfner, Herbert H. T. Prins

**Affiliations:** ^1^ Department of Biological Sciences Centre for Wildlife Ecology Simon Fraser University Burnaby BC Canada; ^2^ Albert Katz International School for Desert Studies Jacob Blaustein Institute for Desert Research Ben‐Gurion University of the Negev Midreshet Israel; ^3^ Wildlife Research Division Environment and Climate Change Canada Delta BC Canada; ^4^ Resource Ecology Group Wageningen University Wageningen The Netherlands

**Keywords:** atmospheric carbon dioxide, nutrient subsidy, *Rubus spectabilis*, seabird islands, stomatal density

## Abstract

Triangle Island on Canada's Pacific coast is home to a large, globally important seabird breeding colony. The shrub Salmonberry *Rubus spectabilis* and tussock‐forming Tufted Hairgrass *Deschampsia cespitosa* together form ~70% of vegetation coverage and contain the vast majority (~90%) of seabird nesting burrows. Salmonberry has in recent decades greatly expanded its coverage, while that of Tufted Hairgrass has receded. Seabirds prefer not to burrow under Salmonberry, making its ongoing expansion a potential conservation issue. We investigated three hypotheses proposed to explain Salmonberry's expansion (climate change, biopedturbation, and nutrient input), using comparisons of stomatal density of Salmonberry leaves sampled from Triangle Island, other seabird colonies, other coastal locations, and from historical specimens in herbaria. Stomatal density helps regulate photosynthetic gain and control water loss, and responds to light, nutrient, carbon dioxide, and water availability. Differing patterns of stomatal density are expected among sample locations depending on which of the hypothesized factors most strongly affects Salmonberry's performance. Our data are most consistent with the nutrient input hypothesis. We discuss possible reasons why Salmonberry has expanded so recently, even though Triangle has been a large seabird colony for at least a century and likely much longer.

## Graphical abstract

Measures of stomatal density made in leaves of the shrub Salmonberry Rubus spectabilis at sites along coastal British Columbia support the hypothesis that nutrient input from the large seabird colony is driving its expansion on Triangle Island. Previous papers on this issue identified “climate change” and “biopedturbation” as the causes. The approach is novel and provides another way to assess vegetation changes on seabird islands.

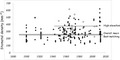

## 1 INTRODUCTION

Ecologists have long been interested in the interactions between seabirds and vegetation on breeding colonies (Duda et al., 2020; Mulder et al., 2011). Seabirds do not graze the vegetation, but their heavy traffic at large colonies can exert effects by digging, trampling, and disturbance, as well as by nutrients in the large quantity of guano deposited. Hipfner et al. (2010) and Rodway et al. (2017) review this topic in papers on the vegetation of Triangle Island, British Columbia, Canada, a large (>1 M individual birds of 11 species) seabird colony. Triangle Island is home to most of the world's Cassin's Auklets *Ptychoramphus aleuticus* as well as a large number of Rhinoceros Auklets *Cerorhinca monocerata*. The shrub Salmonberry and tussock‐forming Tufted Hairgrass *Deschampsia cespitosa* together makeup about 70% of the vegetation coverage and between them contain virtually all nesting burrows of Cassin's and Rhinoceros Auklets. Presumably because its tall shrubby canopy makes access difficult, and its tough and dense root system makes excavation arduous, these species avoid Salmonberry and favor Tufted Hairgrass as nesting habitat (Rodway et al., 2017; Vermeer et al., 1979). The coverage of Salmonberry has greatly expanded in recent decades at the expense of Tufted Hairgrass, raising serious concerns that the suitability of Triangle Island as a breeding colony is shrinking (Hipfner et al., 2010). Indeed, due to concern about habitat changes, both marine and terrestrial, Cassin's Auklet was recently listed as a species of Special Concern by the Committee on the Status of Endangered Wildlife in Canada (COSEWIC).

Salmonberry (*Rubus spectabilis*) is a deciduous perennial shrub of the Rosaceae family, endemic to the Pacific Northwest (Oleskevichl et al., 1996; Tappeiner et al., 1991; Zasada et al., 1994). Salmonberry grows up to 5 m in height, with aerial stem densities of 2–10 m^−2^ woody mature stems bearing compound serrate trifoliate leaves 10–20 cm in length (Oleskevichl et al., 1996; Zasada et al., 1994). The flowers are solitary, deep pink, 2–4 cm wide, and are pollinated by insects and hummingbirds (Oleskevichl et al., 1996). After planting, new salmonberry roots take only a few weeks to begin developing, making them quick to establish compared to other plants (Cowan, 2008; Tappeiner et al., 1991). Salmonberry aggressively colonizes disturbed sites, and outcompetes many other species (Oleskevichl et al., 1996; Tappeiner et al., 1991; Zasada et al., 1994).

Several hypotheses have been proposed to explain Salmonberry's expansion on Triangle Island. Based on data from permanent plots measured at 5‐year intervals 1989–2014, Rodway et al. (2017) concluded that the expansion of Salmonberry has been enabled by declining seabird numbers. Their proposed mechanism is that soil disturbance (“biopedturbation”) by seabirds digging and maintaining nesting burrows negatively affects Salmonberry, reducing its coverage when seabird numbers are high and enabling its expansion when seabird numbers decline. Hipfner et al. (2010) suggested that rising temperature and falling precipitation, evident in weather records collected at the nearby Cape Scott light station, 1970–2004, made conditions more favorable for Salmonberry (Chapuis et al., 2004; Donlan et al., 2003), supporting more vigorous growth and expansion on Triangle Island, as well as on nearby Sartine Island, also a seabird colony.

Alternatively, nutrients delivered from the sea to seabird colonies (in the form of guano, dropped food deliveries, and dead seabirds) can influence vegetation (Anderson & Polis, 1999; Bokhorst et al., 2007; Duda et al., 2020; Ellis et al., 2006; Sanchez‐Pinero & Polis, 2000). Croll et al. (2005) provided experimental and comparative evidence from the Aleutian Islands that vegetation around seabird colonies was altered to grassland from its natural tundra state and reverted to tundra when and where Arctic foxes *Vulpes lagopus* were introduced (for fur). These predators greatly reduced seabird numbers and consequently the nutrient supply. Grassland re‐established when foxes were removed and seabird numbers rebounded.

Any of these mechanisms (biopedturbation, climate change, and nutrient input) could alone or in combination be contributing to the expansion of Salmonberry. Identifying which applies is important due to the conservation status of Triangle Island. Discriminating between these potential causes is also challenging because each is changing, and hence they are easily confounded. Experiments like that of Croll et al. (2005) are rarely possible on the scale of entire seabird colonies, so other lines of evidence to help disentangle the relative contributions of these factors would be useful. To this end, we measured stomatal density in Salmonberry leaves sampled along coastal British Columbia, and from leaves in herbarium collections. Differing patterns of stomatal density among these samples are expected depending on which of the three hypothesized factors affect Salmonberry's performance.

Plants maintain the balance of CO_2_ and nutrient gain with transpirational water loss through pores (stomata) on the leaves of most vascular plants. Stomatal aperture size can be adjusted via the temporary closure of guard cells, and by adjusting the stomatal density of newly produced leaves (Broadley et al., 2001; Evans & Seeman, 1989); (Manzoni et al., 2013). Such phenotypic plasticity has been documented in most plant taxa, and is common in the Rosaceae (Woodward & Kelly, 1995). The stomatal density of many species has fallen over the past century in response to the increasing availability of CO_2_ in the atmosphere (Beerling & Kelly, 1997; Franks & Beerling, 2009; Frey et al., 1996; Luomala et al., 2005; Mao et al., 2005; Morison, 2001; Rivera et al., 2014; Van de Water et al., 1994; Woodward, 1987). Conversely, in relatively low CO_2_ environments, or in environments with excess nutrient or water availability, stomatal density can increase (Frey et al., 1996; Körner et al., 1986; Kouwenberg et al., 2007; Pazourek, 1970; Sáez et al., 2012; Siegwolf et al., 2001).

## 2 HYPOTHESES AND PREDICTIONS

Differing patterns in stomatal density are expected among locales depending on whether increased carbon dioxide availability or other aspects of climate change (e.g., precipitation), nutrient supply, or biopedturbation is the dominant influence. Stated most broadly, the climate change hypothesis is that the recent expansion of Salmonberry at the expense of Tufted Hairgrass on Triangle Island is attributable to one or more of the atmospheric changes in the past century. These include increased carbon dioxide concentration, higher temperature, altered precipitation, and perhaps others such as wind speed or storm intensity. If the increase in atmospheric carbon dioxide is important, we expect the density of stomata to have fallen over the past century, as has been observed in many other species. A systematic, widespread decline would indicate that Salmonberry has responded to the atmospheric rise of CO_2_, buttressing the hypothesis that climate change has contributed to its expansion on Triangle Island. We test this hypothesis with measures of stomatal density in Salmonberry specimens from herbaria collected since 1895 along British Columbia's coastline, as well as in collections made for this study.

The “nutrient input” hypothesis is that the expansion of Salmonberry on Triangle Island is attributable to the heightened nutrient (likely nitrogen) availability due to the guano deposited on the island by the large seabird population. We test the “nutrient input” hypothesis by comparing the density of stomata in leaves collected on seabird colonies of varying size, and on islands without seabird colonies. If the nutrient input is a dominant factor, stomatal densities and seabird colony size should be positively related, with the highest densities on the largest colonies. But if carbon dioxide concentration is the dominant influence, there is no reason to expect any differences.

The “biopedturbation” hypothesis is that the expansion of Salmonberry on Triangle Island is due to the recent reduction in seabird numbers. On Triangle, Pine, and Lucy Islands, burrows are densely packed in the main areas of the colony where leaf sampling took place. Rodway et al. (2017) report burrow densities of 4.3–7.0 per permanent plot (area 100 m^2^). Burrows can be several meters in length and can have several branches. Occupied burrows are actively maintained by digging throughout the lengthy breeding season. A decline in the seabird numbers would reduce the extent of burrowing activity, hypothetically enabling Salmonberry to recolonize areas from which it had previously been eradicated. If biopedturbation is the dominant influence, as hypothesized by Rodway et al. (2017), we expect stomatal density and the size of the colonies to be negatively correlated, as plants adjust to the reduced nutrient and water supply caused by soil disturbance and root damage.

## 3 METHODS

We located 184 Salmonberry specimens during visits to herbaria at the Royal British Columbia Museum, the University of British Columbia, and Simon Fraser University. The specimens span the 20th century, with the earliest dating to 1895. The collection sites are scattered along the B.C. coast. A single leaf was collected at most sample locations, described as, for example, “shoreline, Cumshewa Inlet, Queen Charlotte Islands”; or “Mount Waddington, 2400 m”. We assigned each of these leaves to one of several broad categories, listed in Table 1 (under “herbaria”). Most (180/184) could be assigned unambiguously based on the information in herbarium records, although four specimens had too little information.

**TABLE 1:** List of Salmonberry (*Rubus spectabilis*) leaf samples, from herbaria and contemporary collections

Category	Mean	SD	*N*	Years	Slope	*r*
HERBARIA						
Elevations >1,000 m	377	92	20	1946–2006	−0.80	0.17
Small islands	251	103	20	1944–2004	0.59	0.10
Vancouver Island	270	91	65	1895–1989	0.75	0.26
Haida Gwai'i	273	63	11	1901–1987	1.96	0.66
Coastal B.C.	264	68	18	1922–1997	1.14	0.46
Mainland B.C.	262	91	35	1943–1988	−0.32	0.05
Vancouver city	239	29	9	1922–2011	0.67	0.04
Unassigned	269	64	4			
Triangle Island	498	/	2	1913		
OVERALL	279	95	184	1895–2011	0.5	0.16
BEST MATCH^a^	267	82	142	1895–2011	0.69	0.17
CONTEMPORARY						
Pacific Spirit Park	238	67	20	2012		
Burnaby Mountain	271	75	75	2012		
Noon's Creek	277	111	20	2012		
OVERALL	266	81	115	2012		
SEABIRD ISLANDS						
East Limestone Island	130	35	19	2016		
Lucy Island (high)^c^	259	87	23	2016		
Lucy Island (low)^c^	252	73	20	2016		
Triangle Island^b^	390	105	7	1913, 2011		
Pine Island	493	87	6	2011		

Note

For each is given the mean stomatal density (mm^−2^), the standard deviation (SD), and the sample size (*N*), as well as (the range of) years collected are summarized. For the herbarium samples, the slope and correlation coefficient of the linear regression are given.

^a^ Sample composition best matching the contemporary “overall” category excludes high elevation sites, small islands, and Triangle Island. “Overall” and “best match” means were calculated from the pooled samples.

^b^ The two 1913 herbarium specimens from Triangle Island are included here. Without these, the mean and standard deviation are 347 and 92, respectively.

^c^ The samples from Lucy Island come from dense (“high”) and less dense (low) areas of the breeding colony.

We collected 115 Salmonberry leaves in 2012 at three locations around Simon Fraser University's Burnaby campus, and 73 Salmonberry leaves on opportunistic visits to four seabird colonies 2011–2017 (Table 1). Leaves were collected following the protocol described in Van den Top et al. (2018). We collected healthy, fully expanded mature leaves. Leaves were collected from the same position within a plant (chest height, outer stem) to control for possible intra‐plant variation in stomatal density. To ensure that leaves were collected from different clones (typically less than 5 m in diameter; Tappeiner et al., 1991), the leaves collected at any site were separated by at least 10 m. Each leaf was pressed and dried for 3 days or more before storage.

The stomatal density of each of these leaves was measured using the (non‐destructive) “nail varnish impression” method described by Van Den Dries et al. (2013), Geisler et al. (2000), and Kolodziejek and Michlewscka (2015). Clear nail varnish was applied to the bottom (abaxial) side of a leaf not more than 1 cm from the midvein, at the widest portion of the middle of the three leaflets, between the second and fifth lateral secondary veins on each side of the leaf. After drying for ~20 min, the layer of varnish was removed using clear tape, and mounted on a microscope slide. Photographs of the impressions were taken with a Canon 5D Mark II camera mounted on a Nikon Eclipse 600 microscope equipped with a Nikon Plan Fluor 20x objective and a Nikon 2.5x phototube lens using bright‐field illumination. Multiple focal planes were photographed to generate focused images of the majority of stomata. Images of focal planes were imported into Adobe Photoshop CC, and a black circle was placed on each stoma with each circle on a separate layer. Stomatal dots were then counted automatically with Pixcavator Image Analysis software to acquire the total stomatal number per image.

We followed the method developed by Van den Top et al. (2018) to derive estimates of stomatal density from these counts. Each impression was counted twice, and the counts averaged. The counts from the pair of impressions taken from each leaf were averaged, and that count was divided by the area of the image field (0.1944 mm^2^) to estimate that leaf's stomatal density, expressed as the number per square millimeter. To correct for inter‐observer differences, each of the three observers measured a standard subset of 20 microscope slides, presented in random order, and counted blind. The slopes of the relationships between pairs of observers were statistically indistinguishable from 1.00 (0.99 and 1.02), with high correlation coefficients (0.78 and 0.73). However, intercepts differed between observers, and so counts were converted to a standard by adding offsets to adjust.

## 4 RESULTS

**FIGURE 1:**
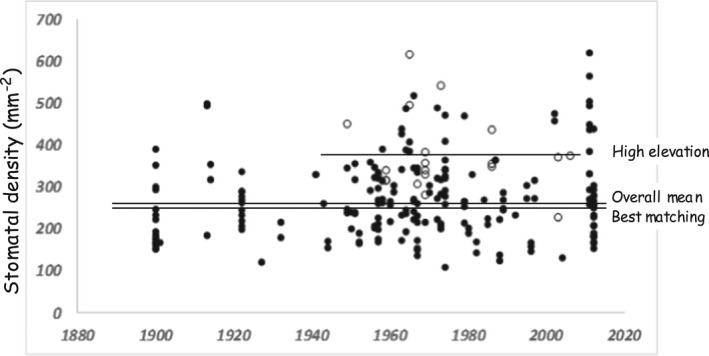
Historical record of stomatal densities (mm^−2^) of salmonberry (*Rubus spectabilis*) specimens collected along coastal British Columbia. Each point represents one specimen (*n* = 372). High elevation specimens (>1,000 m altitude) represented by open dots. The horizontal lines represent the mean stomatal density of (from top to bottom) high elevation specimens (377 mm^−2^), the overall (279 mm^−2^), and the “best matching” (see text; 267 mm^−2^) mainland sets

We measured the stomatal density of 372 Salmonberry leaves (Table 1), displayed in Figure 1 in relation to the year of collection. The overall mean is 279 stomata mm^−2^ (standard deviation 95 stomata mm^−2^), with no long‐term trend evident (overall slope between 1895 and 2011 does not differ significantly from zero). A power analysis calculated that a slope as small as 0.1 would have been detected with 95% probability with the herbarium sample alone (*n* = 182), so the non‐significance is unlikely to be a false negative. Table 1 also reports for seven (of the nine) herbarium categories the slope and correlation coefficient of the change in stomatal density over time. Of these, two slopes are negative and five positive, and none differs significantly from zero. These comparisons provide no suggestion that stomatal density has changed over the past century. The possibility that Salmonberry is incapable of change can be discounted because, as has been found in other species, its stomatal density at high elevation sites (377 stomata mm^−2^; standard deviation 92 mm^−2^) is strongly (by 98 stomata mm^−2^; 35%) and significantly (*t* = 4.735, *df* = 23, *p* < .0001) greater than the overall mean of 279 mm^−2^.

It is possible that the expansion of Salmonberry was driven by changes in temperature or precipitation rather than of carbon dioxide, so that growth vigor was enhanced with the same level of stomata. If precipitation increased over recent decades, stomatal density could remain constant (or perhaps even decrease). Over the period from 1970 to 2005, the Scott Island archipelago (which includes Triangle Island) has become warmer (up by ~1°C from 10.5°C, average air temperature from April to August inclusive) but also drier (down by ~200 mm from 625 mm, precipitation from April to August inclusive; based on weather records at nearby Cape Scott; see Figure 7 in Hipfner et al., 2010). These trends do not support this alternative explanation, as the decrease in precipitation and increase in temperature would both contribute to reducing the water supply.

The overall mean stomatal density of the contemporary “mainland” samples collected in 2012 (see Table 1) is 266 stomata mm^−2^ (standard deviation 81 mm^−2^). The sample locations are all similar sites in the vicinity of Simon Fraser University's Burnaby campus. To create a sample composition from the herbarium collections matching this collection, we excluded high elevation sites, small islands, and Triangle Island. The resultant “best matching” sample has a mean of 267 stomata mm^−2^ (standard deviation 82 stomata mm^−2^), identical to that of the contemporary mainland collection. We use the overall mean of these groups (267 stomata mm^−2^) as the baseline for additional comparisons.

We sampled contemporary Salmonberry leaves from four seabird islands. The stomatal density at the large (100 K–1 M birds) colonies at Triangle (390 stomata mm^−2^) and Pine Islands (493 stomata mm^−2^) both significantly exceed the baseline, by 123 stomata mm^−2^ (47%; *t* = 3.07, *df* = 5.9, *p* = .0126) and 226 stomata mm^−2^ (85%; *t* = 6.25, *df* = 5, *p* = .0003), respectively. The Lucy Island colony (10,000 breeding pairs of Rhinoceros Auklets) is smaller than those on Triangle and Pine Islands. The stomatal density is 256 stomata mm^−2^, which does not differ from the baseline value of 267 stomata mm^−2^, but is lower by 135 stomata mm^−2^ (50.1%) than Triangle Island, and by 238 stomata mm^−2^ (89.1%) than Pine Island (both comparisons *p* < .001). The stomatal density on East Limestone Island (130 stomata mm^−2^) was the lowest measured in any sample, lower by 137 stomata mm^−2^ (51%) than the baseline (*t* = 12.33, *df* = 56.4, *p* = .0000), and lower by 121 stomata mm^−2^ (48%) than other small (non‐seabird) islands in the historical sample (*t* = 4.96, *df* = 23, *p* = .00002).

## 5 DISCUSSION

The data presented here indicate that the mean stomatal density of Salmonberry leaves collected in coastal British Columbia has remained steady since at least 1895, the date of the earliest specimens in herbarium collections. The lack of any temporal trend and the fact that the means of the contemporary and herbarium samples are closely matched do not support the hypothesis that the rise in atmospheric carbon dioxide has been a strong factor in affecting the performance of Salmonberry. We can discount the possibility that Salmonberry is for some reason unable to respond because as found in many other plant species, high‐elevation populations have higher stomatal density.

A possible explanation for the lack of response is that coastal British Columbia is so wet that water conservation is never an issue. This seems unlikely: coastal British Columbia often has pronounced dry conditions in late summer, and we have often noted dead leaves and stems at these times. In their analysis of Salmonberry stomatal density, Van den Top et al. (2018) found that the positive relation of stomatal density to nutrient density is much more pronounced at locations with high soil moisture. Thus, even in the wet temperate rain forests of coastal British Columbia, water conservation appears to be a consideration at least during one part of the year.

The four seabird colony samples display a large stomatal density range, ranging from one half (East Limestone Island) to nearly double (Pine Island) the baseline. The high stomatal density and vigorous growth of Salmonberry on Triangle Island contrast strongly with the low stomatal density and low overall abundance of Salmonberry on East Limestone Island. Precipitation does not appear to be a dominating factor, as these sites have the lowest (East Limestone: 130 mm^−2^) and the second highest (Triangle: 390 mm^−2^) measures in our collections, and yet have about the same annual precipitation (~1,400 mm). If biopedturbation were the prime agent underlying Salmonberry performance, we would expect to measure lower values on larger colonies. However, the rank order of stomatal density on the four seabird islands is consistent with colony size, and thus with a positive response to the availability of nutrients supplied by seabirds.

The low value on East Limestone Island corresponds to the biology of its Ancient Murrelet (*Synthliboramphus antiquus*) breeding colony. These seabirds dig nesting burrows, but chicks are precocial and depart the nest at 1 or 2 days of age. Parents deliver no food to the burrow, and no guano is deposited. This cleanliness perhaps evolved as an antipredation trait (Gaston, 1992), but whatever the cause, their breeding activities provide no nutrient input, while the other attributes of a seabird colony—including biopedturbation—remain intact. In fact, East Limestone Island stomatal density is lower even than on non‐seabird islands. This might be because it is naturally very nutrient poor, has a high level of browsing by the deer endemic to the island, or because burrowing by murrelets negatively affects Salmonberry performance, as suggested by Rodway et al.'s (2017) hypothesis.

Stomatal density levels on Lucy Island (251 mm^−2^) match those reported by Van den Top et al. (2018; ~250 mm^−2^) on the densest salmon‐bearing stream near Bella Bella, on B.C.'s central coast. The size of the nutrient input at these locations appears similar. Van den Top et al. (2018) report the salmon run biomass in this stream at about 15,000 kg per year. We estimate that the Rhinoceros Auklet breeding colony on Lucy Island delivers approximately this amount of fish in a typical breeding season. The ~10,000 breeding pairs of each deliver ~2.5 kg wet weight of fish per season (Bertram et al., 1991). Not all is deposited on the island because chicks fledge at 400–500 g, and there is some breeding failure. The areas of the riparian area along the stream and that of Lucy Island are approximately equal (~10 ha), suggesting that the density of the nutrient subsidy is similar, and forms an important influence on stomatal density.

The large number of seabirds on Triangle Island has been noted in the scientific literature since the early 1900s. The number of seabirds on the colony has if anything recently become smaller (Rodway et al., 2017), and the question therefore arises if the nutrient input is responsible, why Salmonberry has so recently expanded. There are several possibilities, including factors, not considered here. For example, the recently increased presence of Bald Eagles *Haliaeetus leucocephalus* has had strong impacts on seabirds on Triangle Island and elsewhere (Henson et al., 2019; Hipfner et al., 2012) that could propagate to other trophic levels. Another possibility is that the loss of nearby Lanz and Cox Islands as suitable breeding colonies (due to the introduction more than a century ago of the American Mink *Neovison vison* and the Raccoon *Procyon lotor* as furbearers; Hipfner et al., 2010) has so increased the nesting density on Triangle Island that nutrient effects are now especially strong.

**FIGURE 2:**
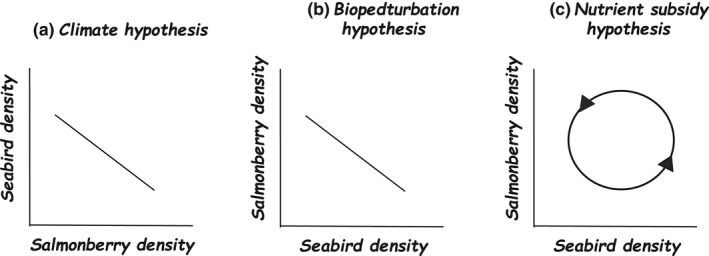
Hypotheses proposed for the recent expansion of Salmonberry on Triangle Island have different implications for the controlling factors. The climate change hypothesis (panel a) implies that salmonberry performance is the independent variable. The biopedturbation hypothesis (panel b) implies that seabird numbers regulate the expansion of salmonberry. The nutrient subsidy hypothesis (panel c) suggests that causation naturally reverses in direction, and that Triangle Island is currently in the upper right quadrant (expanding salmonberry coverage, falling seabird numbers)

The three hypotheses considered here have differing implications for the interaction of seabirds and Salmonberry. The climate change hypothesis implies that Salmonberry performance controls seabird numbers, and it is hence in Figure 2a the independent variable. The biopedturbation hypothesis, in contrast, implies that seabird numbers control the expansion of Salmonberry, which is hence in Figure 2b the dependent variable. The nutrient subsidy hypothesis suggests that causation naturally reverses in direction: due to the low supply of nutrients on which it depends, Salmonberry performs relatively poorly at low seabird numbers, allowing seabird burrows to expand. The subsequent increase in nutrient input supports the expansion of Salmonberry, which reduces seabird numbers and hence the nutrient subsidy, leading to contraction of Salmonberry and enabling seabird burrowing density to rise. This dynamic relationship resembles that suggested by Rodway et al. (2017), who proposed that burrowing seabirds affect the equilibrium between the natural succession (toward a thick continuous cover of Salmonberry) and the open grassy habitat in which seabirds prefer to burrow. These ideas are not incompatible, but differ in which effect is thought to dominate.

In conclusion, this study examined the stomatal density of Salmonberry leaves throughout coastal British Columbia over the last 120 years. No temporal effects were observed, suggesting that increased atmospheric CO_2_ has not had an effect on Salmonberry performance. Salmonberry leaves collected from high elevation sites tended to have higher stomatal density, indicating that Salmonberry stomatal density can be modulated according to environmental conditions. Islands with large colonies of guano‐depositing seabirds tended to have high stomatal density, whereas smaller islands, particularly with birds with tidy nesting behavior (low nutrient input), had lower stomatal density. Such observations suggest that a naturally reversing interaction between nutrient subsidies and seabird nesting behavior contributes to long‐term fluctuations in seabird island—vegetation dynamics.

## AUTHOR CONTRIBUTIONS

**Ron Ydenberg:** Conceptualization (equal); data curation (equal); formal analysis (lead); funding acquisition (lead); investigation (equal); methodology (equal); project administration (lead); resources (lead); supervision (equal); validation (lead); writing – original draft (lead); writing – review and editing (lead). **Ben Leyland:** Data curation (equal); formal analysis (equal); investigation (equal); methodology (equal); validation (equal); writing – original draft (equal); writing – review and editing (equal). **Mark Hipfner:** Conceptualization (equal); funding acquisition (equal); methodology (equal); resources (equal); validation (equal); writing – original draft (equal); writing – review and editing (equal). **Herbert H. T. Prins:** Conceptualization (lead); investigation (equal); methodology (equal); supervision (equal); validation (equal); writing – original draft (equal); writing – review and editing (equal).

## Data Availability

The dataset was archived in Dryad Nov 16, 2021: https://doi.org/10.5061/dryad.ns1rn8pv7.
